# A Case of Necrotizing Fasciitis Mimicking a Burn in an Elderly Patient

**DOI:** 10.1155/2023/3786364

**Published:** 2023-03-25

**Authors:** Madiha Khan, Shubham Bhatia, Kelly L. Cervellione, Martine A. Louis

**Affiliations:** ^1^New York Institute of Technology College of Osteopathic Medicine, Old Westbury, NY, USA; ^2^Flushing Hospital Medical Center, Flushing, NY, USA; ^3^MediSys Health Network, Queens, NY, USA

## Abstract

Necrotizing fasciitis travels along the fascial plane and surrounding soft tissue, leading to ischemia and necrosis. Fournier's gangrene is a type of necrotizing fasciitis invading the deep and superficial planes of the perineal/genital region. It is rapidly progressive in nature and may have life-threatening consequences. Fournier's often exhibits a misleading clinical presentation and can be mistaken for other conditions, such as hematoma, phlebitis, cellulitis, or septic arthritis. Since the ramifications of delayed diagnosis can be clinically significant, recognition of potential mimics is important to prevent morbidity or mortality. We report a case of Fournier's gangrene mimicking a second-degree burn, an exceedingly rare presentation.

## 1. Introduction

Fournier's gangrene is a type of necrotizing fasciitis involving the deep and superficial planes of the perineal genital region [1]. The inflammation associated with Fournier's gangrene moves along the fascial plane and surrounding soft tissue, eventually leading to ischemia and necrosis [1]. Due to its often subtle initial presentation, Fournier's gangrene can be mistaken for other conditions, such as hematoma, phlebitis, cellulitis, or septic arthritis [2]. Left untreated, Fournier's can rapidly progress and lead to significant morbidity or mortality, making timely diagnosis imperative. Here, we report the case of a patient with Fournier's gangrene who presented with a wound in the right perineum mimicking a second-degree burn, an exceedingly rare sign of the disease process.

## 2. Case Presentation

**Figure 1:**
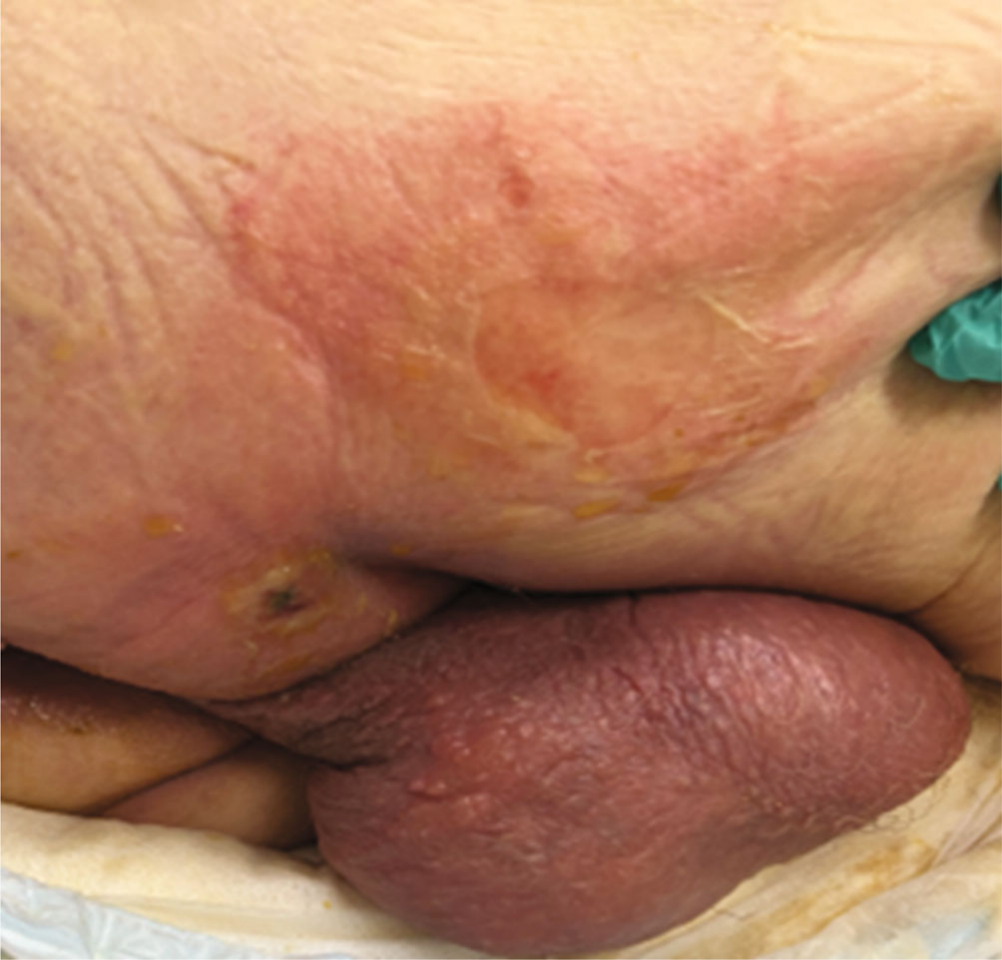
Right gluteal fold with erythema and presence of one ruptured blister with desquamation and one fluid filled blister.

A 77-year-old male with a history of benign prostatic hyperplasia, coronary artery disease (CAD) with stent placement, hypertension, hyperlipidemia, and Parkinson's disease, presented to the emergency department with fever, altered mentation, and a right gluteal wound extending into the perineal region. The family reported that a heating pad had been applied over the area 24 hours prior to the development of the wound because of complaints of pain in the area. On arrival, the patient was diaphoretic. His vital signs were: temperature = 100.1 F, blood pressure = 122/70 mmHg, pulse (HR) = 98, respiratory rate (RR) = 24, and oxygen saturation = 91% on room air. He was placed on supplemental oxygen (4 L nasal cannula) which improved his saturation to 99%. On examination, the perineum and right gluteal folds appeared erythematous with desquamation and blisters but without fluctuance or purulence (Figure 1). This was consistent with a second-degree burn. Labs were significant for white blood cell (WBC) count of 29.1 K/*μ*L (reference: 3.8–11.0 K/*μ*L), lactate of 2.15 mmol/L (reference: 0.7–2.00 mmol/L), and C-reactive protein (CRP) of 13.0 mg/dL (reference: <0.9 mg/dL). Laboratory Risk Indicator for Necrotizing Fasciitis (LRINEC) score was 3 (calculated using MDcalc). Initial treatment included intravenous fluid bolus and vancomycin and piperacillin–tazobactam intravenously, and transfer procedures to a burn facility were initiated.

**Figure 2:**
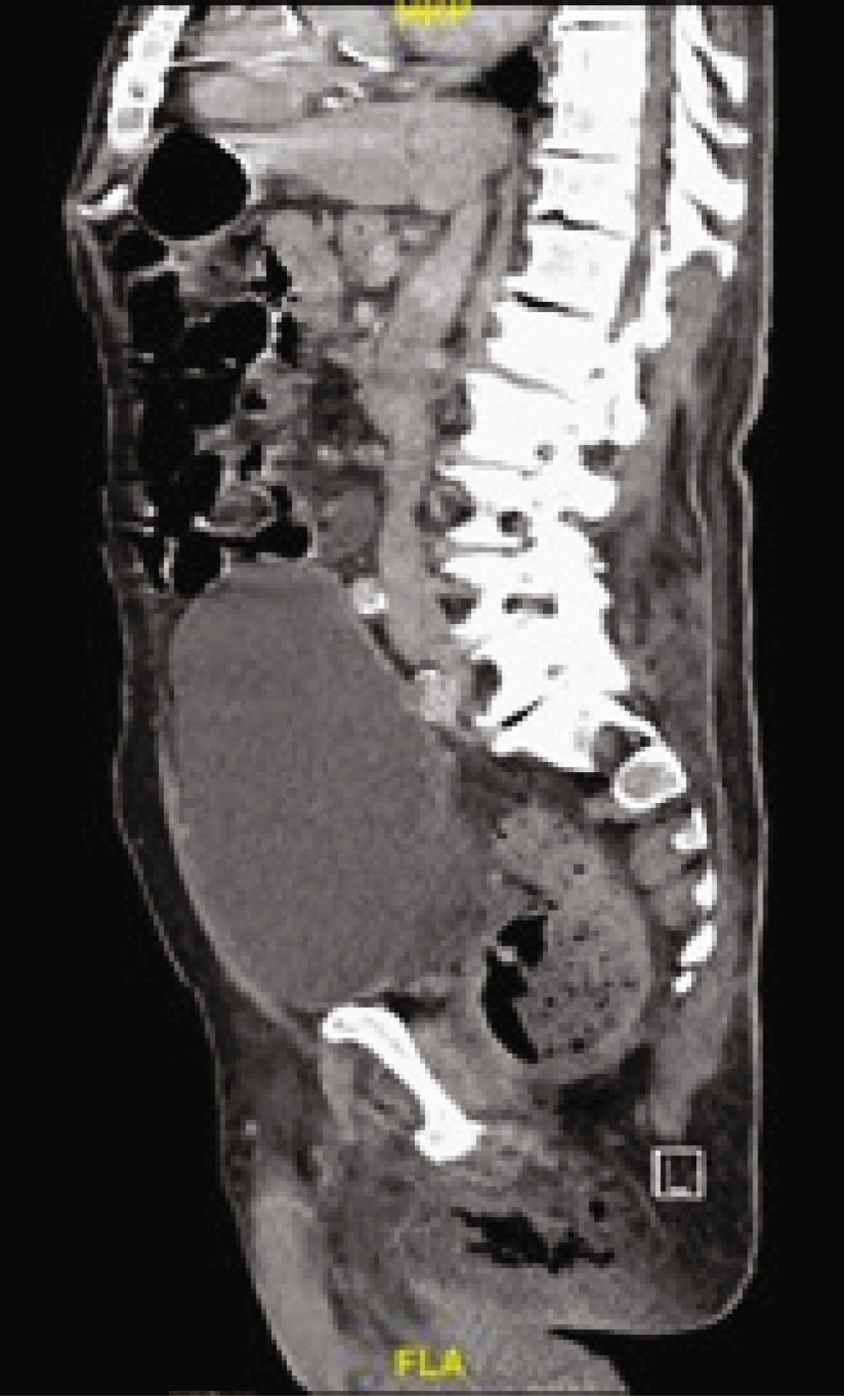
CT abdomen/pelvis illustrating numerous abnormal foci of air involving the tissues of the perineum extending into the proximal right thigh consistent with Fournier's gas gangrene.

A computerized tomography (CT) scan of the abdomen/pelvis had been ordered as the patient waited, but results became available only after the patient's transfer. The CT scan illustrated abnormal foci of air involving the perineum extending into the proximal right thigh with asymmetric thickening of the right spermatic cord, consistent with Fournier's gas gangrene (Figure 2). At the accepting facility, the patient underwent surgical excisional debridement and was intubated along with placement of a wound vacuum. Only one debridement was necessary in this case and the patient was monitored in the surgical intensive care unit for three days before being transferred to the step-down unit. Dressings were changed twice daily with application of bacitracin ointment. Wound cultures grew *Escherichia coli* and *Enterococcus avium*; clindamycin was added to vancomycin and piperacillin–tazobactam. His mildly elevated temperature present on initial visit had resolved and the rest of the vitals remained stable, similar to the above vitals, during his hospital course. However, his postoperative course was complicated by a non-ST elevation myocardial infarction, which was successfully medically managed. He was extubated on postoperative day 2, and was discharged on postoperative day 12. After discharge, the wound was managed with twice daily wet to dry dressings and bacitracin ointment. At 5 weeks post-discharge, he had significant improvement with only a sub-centimeter wound remaining.

## 3. Discussion

With both the physical and financial burdens of disease being high in patients with necrotizing fasciitis, it is imperative to bring more awareness to the various presentations of this disease. The median total inpatient charge for the treatment of necrotizing fasciitis in the United States in 2001 was $54,533 with cumulative charges for all patients reaching nearly $20 million [3]. Furthermore, management of wounds after discharge and changes to physical activity due to the wound can create an additional physical burden of disease for elderly patients who are likely to have other comorbidities as well.

## 4. Pathophysiology

Necrotizing fasciitis is a life-threatening condition secondary to a deep soft tissue infection [1]. Anaerobic and aerobic bacterial infections from sources, such as the urinary tract or from a perineal abscess incites the development of Fournier's gangrene. The bacterial infection results in subcutaneous vessel microthrombosis (obliterative endarteritis), which progresses to gangrene with the release of bacterial endotoxins and enzymes [1]. Since the process begins in the deep soft tissue, early skin manifestations may be absent as the necrosis may not have spread to the superficial plane [1]. This contributes to the diagnostic challenges in the initial stages of necrotizing fasciitis, as it can mimic other conditions, such as hematoma, phlebitis, cellulitis, or septic arthritis.

When extension occurs, necrosis spreads not only to the fascia, but also to muscle, skin, and adjacent soft tissues as well [4]. Thus, necrotizing fasciitis involves a cyclical process of life-threatening infection, toxin release, activation of cytokines, microthrombosis with ischemia, and tissue decay [4]. Exotoxins produced by bacteria function as superantigens. They bind antigen-presenting cells leading to T-cell propagation and further cytokine release, exacerbating organ dysfunction, and shock [4]. Because the decaying tissue is poorly penetrated by antibiotics, emergent surgical debridement is essential to obtain source control. Debridement reveals “dishwater fluid” and “positive finger sign,” describing a lack of resistance when dissecting fascial planes with a finger inserted into the plane [4].

Although they may appear similar at times, burns have a different pathophysiology than necrotizing fasciitis. Coagulative necrosis of underlying tissue and layers of skin is initiated by a burn injury [5]. The extent of damage depends on the energy of the causative agent, the time of exposure, and temperature of exposure [5]. Causes of thermal burns include fire, scald, and contact with hot/cold objects. Because the initiating injury is from contact with the skin, burn wounds expand from the superficial skin to the deeper layers as opposed to necrotizing fasciitis [5].

Necrotizing fasciitis is associated with comorbidities, such as diabetes mellitus, hypertension, malignancy, and alcohol abuse [1]. Elderly male patients, those with chronic renal failure, peripheral vascular disease, or immunosuppression are at higher risk of developing necrotizing fasciitis, as in the case of our elderly patient with hypertension, hyperlipidemia, and CAD [2]. Necrotizing fasciitis can be caused by numerous factors, including trauma or recent surgery [2]. Even innocuous causes of trauma (laceration or abrasion) can lead to necrotizing fasciitis [2]. Fournier's gangrene can also be provoked by other sources of infection, for example, urinary tract or bowel infections. In patients who have sustained third-degree burns to the perineum or scalding injury to the lower extremity, development of necrotizing fasciitis within days of the injury has been reported [6, 7].

The microbiome implicated in necrotizing fasciitis is commonly polymicrobial, aerobic, and anaerobic [1]. Group A *Streptococci* and *Staphylococcus aureus* are most commonly isolated. For Fournier's gangrene, specifically, Gram-negative bacteria, *E. coli* and *Pseudomonas aeruginosa*, are also most often implicated.

## 5. Diagnosis

The timely identification of necrotizing fasciitis is challenging in many cases, making it essential to obtain a complete workup, as delayed diagnosis increases the likelihood of morbidity and mortality secondary to rapid deterioration. Recommended labs include a complete blood count (CBC) with differential, comprehensive metabolic panel (CMP), blood cultures, CRP, and lactate level [1]. The CBC can demonstrate an elevated WBC with a left shift indicating an infectious process. CMP can demonstrate derangements such as hyponatremia and hyperglycemia. Blood cultures and a lactate level can determine if there is a presence of sepsis or bacteremia. Wound cultures allow targeted antibiotic therapy. The LRINEC score is a simple score based on WBC, CRP, hemoglobin, creatinine, sodium, and glucose levels [8]. This tool can help clinicians in determining the risk of necrotizing fasciitis, but should not be the sole indicator of necrotizing fasciitis. Our patient had a LRINEC score of 3, associated with <50% risk of the presence of necrotizing soft tissue infections [8]. However, the results of a meta-analysis have called into question the diagnostic accuracy of the LRINEC score, citing poor sensitivity for necrotizing soft tissue infection (LRINEC >6 sensitivity of 68.2%) [9].

Adjunctive imaging is crucial to prevent a missed diagnosis, especially in cases of a low LRINEC score. Appropriate imaging modalities for identifying underlying soft tissue subcutaneous air include ultrasound, X-ray, and CT scan. CT imaging is the gold standard since it has a sensitivity of 88.5% for detecting subcutaneous air in the soft tissue, compared with X-ray that has a sensitivity of 48.9% [9]. CT scan can clearly define the perineal anatomy, demonstrating thickening of the fascia, subcutaneous air, or fluid collection and delineate the extension of the disease process into the retroperitoneum [10].

The differential diagnosis of Fournier's gangrene is broad. If presenting with acute scrotal pain, the differential includes testicular torsion and acute epididymitis. When erythema and edema are present, conditions, such as cellulitis, perianal/periurethral abscess, and gangrenous balanitis, may be suspected. Ulceration can indicate syphilis, chancre, or herpes simplex. A rapidly progressive desquamation can mimic toxic shock syndrome, toxic epidermal necrolysis, and Stevens–Johnson syndrome [1].

On review of the literature, one case report was identified describing necrotizing fasciitis of the forearm mimicking a full-thickness burn in a patient who had sustained a dorsal forearm laceration after a recent fall [2]. Initial evaluation by a plastic surgeon indicated that the wound exhibited the characteristics of a full thickness burn. The patient's rapid deterioration and the quick progression of the wound to deeper tissue prompted reevaluation resulting in a delayed diagnosis of necrotizing fasciitis. Clinically, the lesion in our patient's case was suspected to be a second-degree burn as the history of a heating pad placed on the area seemed consistent with the appearance. However, only a day had passed from the time of the heating pad placement and presentation to the hospital. It was more likely that the underlying inflammation led to the development of necrotizing fasciitis. A change in mental status and elevated WBC can also be observed in burn patients, so these findings may not initially be considered suspicious for necrotizing fasciitis [11].

## 6. Management

The treatment for Fournier's gangrene requires both medical and surgical involvements. First and foremost, fluid resuscitation either with or without vasopressors is used to mitigate the accompanying sepsis [1]. Electrolyte abnormalities may need to be corrected, and empiric broad-spectrum antibiotics are essential. Antibiotics used include a third-generation cephalosporin or aminoglycoside with the addition of penicillin and metronidazole [1]. Some regimens include carbapenems or piperacillin tazobactam [1]. Urgent surgical debridement is always required with wide resection of necrotic gangrenous tissue and often additional excisions may be required; reconstructive surgery may also be indicated. Necrotizing infections originating from the anorectal area involving the anal sphincter or rectal perforations with extensive perineal involvement require a diverting colostomy [1]. In addition to the above management, some authors have suggested the use of low doses of radiation for the treatment of necrotizing fasciitis; however, this modality needs additional research to elucidate its potential [12].

Prognosis of patients with necrotizing fasciitis varies. Tools, such as the Uludag Fournier Gangrene Severity Index (UFGSI), Surgical Apgar Score (SAS), and age-adjusted Charlson Comorbidity Index (ACCI) can be utilized to achieve a prognostic classification. The UFGSI takes into account the patient's age, temperature, heart rate, RR, serum electrolytes, creatinine, bicarbonate levels, hematocrit, WBC count, and extent of tissue affected. Electrolyte abnormalities, such as elevated calcium and low magnesium, are associated with a worse prognosis [1]. ACCI assigns points to various comorbidities with each one assigned a certain score. A higher score correlates with an increased risk of mortality. The 10-point SAS is a perioperative communication tool, which helps predict the 30-day major complications or death after surgery. It is a measure of intraoperative hemodynamic stability that uses estimated blood loss, lowest mean arterial pressure, and lowest heart rate in its scoring schema. A low SAS correlates with a higher chance of complication. The SAS and ACCI are not specific to Fournier gangrene, but are comparable with UFGSI for anticipating prognosis [1].

Time to surgical treatment also affects the prognosis. Earlier presentation and prompt debridement are associated with a better prognosis. Even with surgery, necrotizing fasciitis has a mortality rate of 20–40% [2]. Comorbidities, such as diabetes with hemoglobin A1C >7, are also associated with a worse prognosis [1].

## 7. Conclusions

This case highlights the necessity of high clinical suspicion for necrotizing fasciitis, especially in those with risk factors, such as those who are elderly, male, have diabetes mellitus, are immunocompromised, or are trauma victims. Additionally, it is vital to recognize the innocuous presentation of necrotizing fasciitis in some cases, which can be misleading. A good history, high index of suspicion, thorough exam, complete laboratory work-up, and timely imaging are all required for a quick and accurate diagnosis. Urgent debridement and prompt resuscitation with initiation of broad-spectrum antibiotic therapy are essential in reducing morbidity and mortality from this life-threatening condition.

## Data Availability

No data to report.
